# Eosinophilic Myocarditis: Clinical Case and Literature Review

**DOI:** 10.5935/abc.20180089

**Published:** 2018-07

**Authors:** Paulo Dinis, Rogério Teixeira, Luís Puga, Carolina Lourenço, Maria Carmo Cachulo, Lino Gonçalves

**Affiliations:** Centro Hospitalar e Universitário de Coimbra, Hospital Geral, Coimbra - Portugal

**Keywords:** Eosinophilia, Myocarditis, Hypereosinophilic Syndrome / mortality, Hypereosinophilic Syndrome / drug therapy, Magnetic Resonance Imaging

## Introduction

Eosinophilic myocarditis is a rare and potentially lethal disease characterized by eosinophil infiltration of the myocardium.^1^ The association between eosinophilia and myocardial injury is well established and may present several etiologies, from hypersensitivity and autoimmune diseases to neoplasias and infections.^1,2^ In some cases the etiology remains unknown, and it is denominated idiopathic hypereosinophilic syndrome. Clinical manifestations present a wide spectrum, ranging from mild symptomatology to severe symptoms such as retrosternal pain, rhythm disturbances, and sudden death.^2,3^ The definitive diagnosis is made through endomyocardial biopsy.^1^ Cardiac magnetic resonance imaging is a valid alternative, identifying the main structural changes caused by myocarditis.^4^ Treatment includes neurohumoral therapy, management of cardiac complications, and in cases selected, systemic corticosteroid therapy.^5^ Next, we present the case of a patient with symptomatology suggestive of myocardial infarction, but who in the course of the investigation had the diagnosis of eosinophilic myocarditis.

## Case report

Patient 79 years old, female, who came to the Emergency Department with complaints of epigastralgia with two weeks of evolution and aggravation last night. She denied another accompanying symptomatology. As personal background, she presented unmedicated dyslipidemia and intrinsic asthma with onset in adulthood. She was medicated with bronchodilators and an association of a B2-agonist with inhaled corticosteroids at low doses.

The objective examination showed tachycardia, confirmed on electrocardiogram with sinus rhythm of 125 beats per minute. Analytically had leukocytosis (13.2 x 10^3^/uL) and eosinophilia (2.8 x 10^3^/uL or 23%), C-reactive protein (0.8 mg/dL) and elevation of markers of myocardial necrosis (troponin I of 7.6 ng/mL). Transthoracic echocardiography revealed severe left ventricular systolic dysfunction with an ejection fraction estimated at 30-35%, ventricular septal hypocontractility and an increase in the concentric thickness of the ventricular walls.

Valvular disease was not evident. It was placed as a first hypothesis that it was an acute coronary syndrome, so anti-ischemic therapy with double platelet antiaggregation, enoxaparin, was started and the patient was assigned to an invasive strategy. Coronary angiography did not reveal epicardial coronary disease. After this, the diagnosis of eosinophilic myocarditis in a patient with a known atopic component was likely. She was admitted to hospital for treatment and study. Neuro-humoral, beta-blocker and diuretic therapy were initiated, maintaining aspirin.

**Figure 1:**
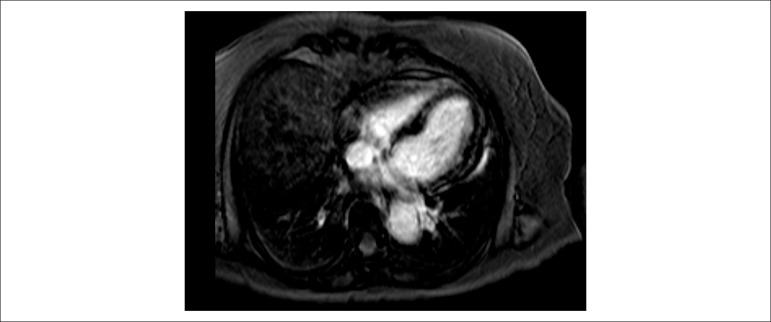
Cardiac magnetic resonance with subepicardial foci of edema and late enhancement of the myocardium in the left ventricle.

**Figure 2:**
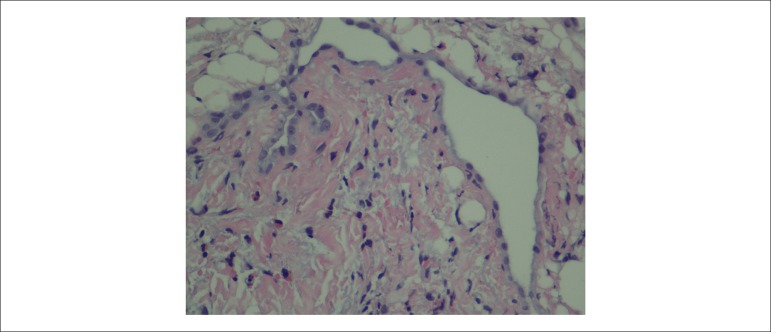
Endomyocardial biopsy with presence of eosinophils.

On the third day of hospitalization, cardiac magnetic resonance was performed which identified subepicardial foci of edema and late enhancement in the left ventricular myocardium (Figure 1); she also showed a small pericardial effusion in the free wall of the right ventricle. The ejection fraction was quantified by 33%. On the same day, she underwent an endomyocardial biopsy and collection of right ventricular infarct fragments, which confirmed the diagnosis of eosinophilic myocarditis (Figure 2). Systemic corticosteroid therapy was started with intravenous prednisolone (1 mg/kg/day) with progressive improvement of general condition. On the 12th day of hospitalization, the echocardiogram showed a slight improvement in left ventricular global systolic function (ejection fraction estimated at 35-40%). She was discharged to home with prednisolone in weaning, and with follow-up consultation of cardiology and autoimmune diseases.

The autoimmune serological study was negative. After seven months of corticotherapy, the echocardiogram showed a significant improvement (ejection fraction estimated at 45-50%), and a decrease in concentric hypertrophy.

## Discussion

In the case described, the patient had a history of asthma, which may have been the starting point for hypereosinophilia. She also presented an epigastric discomfort, which may be an atypical presentation of an acute coronary syndrome.^6^ Electrocardiographic findings, sinus tachycardia, are neither specific nor sensitive.^1^ Analytically, leukocytosis and eosinophilia with troponin I elevation were evident and explained by infiltration of eosinophils into the myocardium. This infiltration allows the release of toxic granules, cationic proteins, pro-inflammatory cytokines, and oxygen free radicals that will cause mitochondrial dysfunction, myocyte injury and necrosis.^7^

Complementary diagnostic tests are important in the evaluation of this pathology. The echocardiogram allows excluding other causes, to monitor dimension of cavities, thickness of the ventricular walls, presence of pericardial effusion and to evaluate left ventricular systolic and diastolic function. Cardiac magnetic resonance provides a combination of safety, anatomical definition, and tissue characterization of the myocardium.^4^ It allowed the identification of edema and diffuse foci of late enhancement reflecting myocardial necrosis and fibrosis.^4^ The presence of pericardial effusion and left ventricular systolic dysfunction reinforce the evidence of myocarditis. In stable patients, it is reasonable to perform cardiac magnetic resonance imaging prior to biopsy, since the former may help to identify focal pathology through late enhancement. However, in unstable patients the biopsy should be a priority.^1^ Endomyocardial biopsy is the only method that allows definitive diagnosis and identification of the underlying etiology. It has a sensitivity estimated at 50% due to sample errors.^1,2^ Although it is the gold standard, in clinical practice it is not always performed, existing recommendations^1,8^ for its execution, which are dependent on the clinic and the results of the complementary tests. The pseudo-ischemic presentation of the patient, with elevation of markers of myocardial necrosis and exclusion of epicardial coronary disease, and alterations in the imaging tests done, fulfilled the criteria for performing the biopsy.^1,8,9^ In these cases, magnetic resonance imaging cardiac and endomyocardial biopsy together present synergies that go beyond the limitations that each exam presents separately.^9^

The treatment and prognosis of eosinophilic myocarditis depends on its etiology. In the acute phase, restriction of physical activity is important.^1^ In selected patients, particularly those with negative virology and suspected autoimmune etiology, early treatment with corticosteroids has shown favorable results.^5,10^ Due to the clinical and hemodynamic stability of the patient, and after infective exclusion, we decided to postpone the onset of corticosteroids until confirmation of eosinophilic myocarditis. In the literature, it is described that a period of immunosuppressive therapy of six months can bring significant improvements in the left ventricular function (increase of 15-20% on the ejection fraction),^10^ which was verified in this case. The question remains whether this improvement is due only to the corticosteroid or if it is associated with initiation of therapy for heart failure, in particular beta-blockers. The mechanism of action of corticosteroids in myocarditis is not fully understood, however it is thought that they interfere with eosinophilia; antagonize the development and maturation pathways; and promote the redistribution of peripheral blood eosinophils.^10^

During follow-up, all patients should be submitted to clinical evaluations with electrocardiogram and echocardiogram. If clinical or imaging worsening occurs, hospital readmission and repeat cardiac magnetic resonance and / or endomyocardial biopsy may be required.^1,9^

## Conclusion

Eosinophilic myocarditis is a rare, undiagnosed condition that can be fatal if not detected and treated in time.
